# Rehabilitation coordinator – managers’ experiences of a new function in health care

**DOI:** 10.1186/s12913-024-11856-6

**Published:** 2024-11-09

**Authors:** Catharina Strid, Rosie Benner, Ronja Stefansdotter, Kjerstin Stigmar

**Affiliations:** 1 Department of Psychology, Lund University, Lund, Sweden; 2 Department of Psychology, Lund University, Lund, Sweden; 3 Department of Psychology, Lund University, Lund, Sweden; 4 Division of Physiotherapy, Department of Health Sciences, Lund University, Lund, Sweden; 5 Department of Psychology, Lund University, Box 213, Lund, 221 00 Sweden

**Keywords:** Managers, Rehabilitation coordinator, Health care, Sick leave, Return to work, Implementation, Qualitative research

## Abstract

**Background:**

Several actions have been taken to improve the sick listing process, patient safety and return to work. One of them is the implementation of the rehabilitation coordinator function, of which the benefits have not yet been fully explored. Neither has the role of the manager, who has significant impact on the implementation and support of a new function. This study aimed to explore how first line managers’, who employed a rehabilitation coordinator that had completed a one-year specialized study program, perceived, and experience the function rehabilitation coordinator.

**Methods:**

This is an interview study using a semi structured interview guide for data collection and thematic analysis was applied to the data. Ten first line managers in health care were interviewed.

**Results:**

Four themes were identified: *The Saviour*,* A personalized function*,* Change takes time and Strengthen status and legitimacy.* The managers experienced the rehabilitation coordinator as a valuable function who facilitates collaboration in the team and with external stakeholders and perceived them as a much-needed resources, supporting physicians with sick leave issues. The assignment was ambiguous and dependent on the rehabilitation coordinators individual characteristics, which may result in a risk of overload. The managers were engaged in the implementation process, but this required time. They considered it important to strengthen legitimacy for the function which required support and encouragement to take part in specialized education and training.

**Conclusions:**

The managers experienced the rehabilitation coordinators as playing a crucial role in the return-to-work process. They were willing to support how this new function will improve and develop. The results from this can serve as a guidance for the implementation and support of the function rehabilitation coordinator.

**Supplementary Information:**

The online version contains supplementary material available at 10.1186/s12913-024-11856-6.

## Background

Rehabilitation coordinator is a function in health care who’s main assignment is to coordinate the return to work (RTW) process for patients on sick leave, by effective coordination and optimizing the length of the sick leave period. Mental disorders and musculoskeletal pain are the most common reasons for patients being on sick leave [1–3]. Health care lack effective interventions for these patients and well-established treatments such as cognitive behavioural therapy and multimodal rehabilitation have shown limited results in relation to sick-leave and RTW [4, 5]. Sick leave certificates are issued by physicians who have reported that sick leave issues compose a significant workload burden. They found the concept of work ability as vague and the assessment time consuming. The lack of instrument made it hard to support the assessments with objective findings and, also difficult to present a prognosis required from the patient and authorities [6, 7]. Based on the sick leave certificate, the Swedish Social Insurance Agency is responsible for deciding about sick leave benefits [8]. Different stakeholders are involved in the sick listing, and it is therefore important to coordinate the RTW process.

In different countries, several actions have been taken to improve the RTW process and decrease sick leave rates such as engage RTW coordinators or implement workplace interventions [9, 10]. In 2019, the Swedish parliament decided on a new legislation regarding rehabilitation coordination [11], which implied that all health care units, both primary and specialized, must offer such coordination services to patients on sick leave. To meet these demands several health care units have assigned a rehabilitation coordinator. The services in rehabilitation coordination include three general areas: (a) internal coordination and collaboration in health care, (b) individual support to the patient, and (c) external collaboration with important stakeholders such as employers and authorities. The fact that the RTW work involves several internal as well as external stakeholders has complicated the improvement of the RTW process [12], and emphasised the need for rehabilitation coordination [13, 14].

Previous studies have been conducted on the benefits of coordinating the RTW process, but they have shown divergent results. Svärd and colleagues (2021) found that patients perceive rehabilitation coordinators as an important resource in coordinating their contacts with different stakeholders, giving them advice and facilitate their communication with their employer [15]). In a Danish study, where patients were interviewed, they found that a lack of coordination led to less treatment of pain and common mental disorders and a feeling of being lost in the health care system [16]. In relation to RTW, Franche et al. (2005) found workplace based RTW interventions such as contact between healthcare provider and workplace, work accommodation and presence of a RTW coordinator to be cost-effective in the short term, but more unsure in the long run [17]. In a systematic review of RTW coordinators effect on RTW, Dol et al. (2021) found strong evidence that face-to-face meetings between patient and coordinators shorten the sick leave duration and increased RTW-rates. Important interventions for the RTW coordinator in these meetings were to identify facilitators and barriers for RTW [18]. A Cochrane review, conducted some years earlier, did not support these findings [19]. There is lack of studies with other type of outcomes and perspectives.

Rehabilitation coordinators or RTW coordinators can be located at workplaces, insurance, or governmental agencies and in clinical settings, and encompass a diverse professional background such as nurses, physiotherapists, occupational therapists, or medical social worker in general [20]. In Sweden, most primary health care (PHC) and psychiatric units have a rehabilitation coordinator, assigned by the manager, who also decide about the extent of the assignment. The assignment is complex and involves several demands such as responsibility to track recording of all patients on sick leave, to be informed and understand the legalisation connected to RTW processes, coordinate RTW activities, collaborate within the health care unit and with different external stakeholders. Health care professionals are not in general trained for this and the tasks require specific knowledge. Therefor management strategies that support and encourage the rehabilitation coordinator to take part in specialized study programs or supervision for the assignment to develop skills such as problem-solving and communication and knowledge about RTW processes and play an important part in the implementation process of the role [20–22]. To meet these demands, different employers and universities have offered both basic courses and further specialized education.

The function rehabilitation coordinator may contribute to organizational changes in health care, and it is therefore important for managers to have a commitment in this change process and to do their utmost to support an innovative working climate [23, 24]. However, guidance for managers how to lead this function, and to know what competencies they require is sparse. Therefore, it was of interest to study how a sample of first-line managers perceive this new function and what experiences they have in leading, developing and working with rehabilitation coordinators.

The aim of this study was to explore first line managers’, who employed a rehabilitation coordinator that had completed a one-year specialized study program, perceptions, and experiences of the function rehabilitation coordinator.

## Methods

### Study design

The study design is based on a qualitative approach. We conducted semi-structured interviews with health care managers,, and applied thematic analyses to the data. To achieve trustworthiness COREQ checklist was used as a support when reporting the study [25], (see Additional file 1).

### Study participants and recruitment process

We contacted all first line managers in a region in southern Sweden who employed a rehabilitation coordinator that had completed a one-year specialized study program (*n* = 24). The one-year program which all rehabilitation coordinators connected to this study attended, was organized by the health care region in the southern Sweden in collaboration with Lund University. Six of the managers who were contacted, replied positively and after sending a reminder two weeks later, another four were willing to participate. No reasons for not participating were reported. In total ten managers, eight women and two men, were included in the study. Their ages ranged from 38 to 61 years old and their experience of being a manager varied between six months and up to ten years. Eight were managers in PHC and two were managers in out-patient psychiatric care. The PHC-centres were in urban areas as well as smaller communities. The PHC-centres differed relative to how many employees and listed patients they had; from the smallest with 15 employees and 3 700 listed patients to the largest with 90 employees and 15,900 listed patients. The two psychiatric care units were general psychiatric out-patient care units, both in middle-sized towns. Before the start, all participants received oral and written information about the study and signed an informed consent.

### Education

The one-year specialized study program was organized by a health care region in southern Sweden in collaboration with Lund University. The program was divided into two parts: (a) first semester the university provided a training course of 7,5 credits in rehabilitation coordination, (b) second semester the health region provided five seminars on different themes parallel with small-group supervision on the assignment held by a licensed psychologist. The themes covered both patient related aspects such as gender, domestic violence and living habits and organizational aspects as leadership, organizational prerequisites, and patient safety. The one-year specialized study program was summarized with a final seminar and examination. Before entering the specialized program, the rehabilitation coordinators participated in a two-day introduction course and had experience of working with the assignment as rehabilitation coordinator.

### Data collection and procedure

The informants that had accepted to participate (*n* = 10) were booked for an interview. All interviews took place in February and March 2019 at the participants’ workplaces. Two of the authors performed five interviews each (RB, RS). The participants were informed that the study was a project for a masters’ degree in psychology and would render in a scientific publication. The interviews were recorded and lasted 50 to 75 min. The interviews were conducted using a semi-structured interview guide to gain a detailed account of the participants’ experiences and beliefs on the topic (see Additional file 2). The interview guide was developed for this study by all four authors. The questions were divided into four sections; (a) comprehensive information about the work place, the rehabilitation coordination and the managers own role (b) deeper into the function of rehabilitation coordinator and the impact of the study program the coordinators had taken (c) deeper into the managers own role in relation to the function of rehabilitation coordinator (d) the participants had the possibility to ask questions and add things to the interview. Before the interviews were conducted, the guide was tested in pilot interviews to ensure that the questions were relevant and that the interviews were conducted in a similar way. After the pilot testing, some minor changes were made in the interview guide, deleting a few questions and re-structuring the four sections to obtain flow in the interview. The recorded interviews were transcribed verbatim and anonymized but were not sent back to the informants for comments.

### Analysis

The analysis was assumed after all interviews had been transcribed to minimize the risk of the writers being influenced by growing patterns of meaning during the latter interviews.

An inductive approach and thematic analysis as devised by Braun & Clarke (2006) was applied to data [26]. The chosen approach was based on the aim that patterns and themes were to be grounded in the data and not from a preconceived framework.

The analysis followed Braun & Clarke’s six steps, which entailed in-depth, methodical listening, writing, and reading to identify patterns of meaning to make codes and finally themes. The program NVivo 12 was used during coding to facilitate thorough searching and to have an overview of a vast amount of data [27]. The analysis was guided by the requirement that patterns of meaning capture something succinct in relation to the aim and not by the prevalence of them. The analysis process meant constantly moving between the data set as whole, individual codes and the themes themselves. Two authors (RB, RS) identified important meanings and labelled these with codes. The codes were then discussed between all four authors and codes were merged to form themes. The process went back and forth to ensure that the different themes were represented in data. All four authors were engaged in the analysis process and accepted the results. Quotes were selected to illustrate the themes.

Along the study the reflexivity was discussed which made the researchers aware of the different personal assumptions and expectations in relation to the aim, methods, and results which in return minimized the risk of personal bias. All four authors are women. Two of the authors are university lecturers and senior researchers (CS, KS) and the other two were, at the time for the interviews, masters ‘students in psychology (RB, RS). The last author (KS) is an associate professor and senior lecture with long experience from conducting interview studies and applying qualitative analysis methods. The first author (CS) is a licensed psychotherapist and a senior lecture in clinical psychology. Both senior researchers (CS, KS) are well familiar with the phenomenon studied, both in clinic, education, and research. Both these authors were engaged in the one-year specialized study program.

## Results

The results describe how first-line managers perceived and experienced the function of rehabilitation coordinator and the possible benefits of their one-year special education. The following four themes were identified during analysis: *The Saviour*,* A personalized function*, *Change takes time* and *Strengthening status and legitimacy.*

### The saviour

Handling sick leave was described as a heavy and cumbersome task that previously has put both patients and healthcare professionals (i.e., physicians) in a state of distress. This was seen as a clear sign that a new strategy was needed which required clarification and a collaborative approach. The managers emphasized the importance of internal and external collaboration and the demand for development of competency in this field. The positive effect of having a rehabilitation coordinator in place was discussed in relation to the reasons why this work was difficult before the position was introduced. Throughout the interviews, the rehabilitation coordinator was described as a saviour in distress.

*“But then I also know that this is an incredible respite for the general practitioners. To not have to feel that they are carrying the whole heavy backpack themselves and make all the decisions by themselves (…).*,* so I know that the practitioners who use the coordinator think it is incredibly valuable and it feels like there is a significantly better quality of what we do.”* Interview 4.

*“After all*,* there are those who are so sick that they simply can’t cope*,* they can’t be bothered to pick up the phone or they have difficulty interpreting the sick leave policy*,* for example (…) I think the rehabilitation coordinator has a huge task to be the one who helps and who is available for questions”* Interview 3.

The managers indicated that the rehabilitation coordinator contributed to increase patient safety. They described that the rehabilitation coordinator contributed to the teamwork and served as a partner in the RTW process not just for the physicians but for the other co-workers as well. The managers emphasized the importance of collaboration around complex cases and regular meetings where the team could discuss patients’ sick leave was described as fruitful. They emphasized that the continuity in the RTW process that the rehabilitation coordinator stands for improved the work environment and made it easier to develop a useful policy. Several of the managers expressed that thanks to the rehabilitation coordinator’s work they were now able to offer a continuity and a plan for patients with longer sick leave.

*“The doctors have a patient from the beginning*,* and then it becomes automatic when you have your rehabilitation coordinators meetings every other week*,* (…) but here you have a patient who is on sick leave for three months*,* what does the plan look like there? (…) It has really been rewarding to sit down in teams and talk (…) Sometimes we just sit around a table and we can solve as much as we want.”* Interview 6.

The coordinator was someone that promoted and fostered collaboration and improved the relationship with external participants. The managers described the coordinators as solving the difficulties the external parts had to collaborate, not due to lack of will but to insufficient communication paths. The coordinator was ultimately seen as someone that relieved the heavy burden of handling the sick leave process. Furthermore, the informants highlighted that the position increased awareness regarding handling sick leave for every part of the chain involved in this complex process. Finally, the unanimous message was that their rehabilitation coordinators had contributed towards the main task of the healthcare system, namely, to strive towards offering safe, sound, just and patient centred care.

*“We have added an extra link in this whole chain*,* and we now have the time needed to get a deeper insight in this than we’ve had before*,* so we are able to ensure quality on a different level. And in my opinion*,* that’s what the biggest change has been*,* and the actual hub of it all.”* Interview 4.

### A personalized function

It became apparent that it was often difficult to separate the role or function from the person that held the position. As reported by the managers, all the rehabilitation coordinators had another profession e.g. counsellor, or physiotherapist so there was a divided focus in their duties. When the position as rehabilitation coordinator was filled and developed, the managers deemed it crucial that the person was suitability for the task and had a strong personal interest in the area. According to several managers, to have a strong interest in sick leave issues was of decisive importance for the function to turn out well. Most of the managers described that their rehabilitation coordinators possessed characteristics suitable for the job, not so much based on their profession but on how they managed the task.

*“She is basically made for this because she knows what she wants*,* has a mind of her own*,* can manage the workplace*,* can identify questions that has to come to light and be worked with*,* for this is a very large assignment.”* Interview 8.

The fusion of the person and the function, the personalized function, meant that a long sought after and now critical position existed solely in one person. This created a potential vulnerability for both the organization/workplace and the rehabilitation coordinator themselves. No one did the work in case of absence. The managers also expressed concerns about the coordinators holding two positions, the rehabilitation coordinator assignment and their profession, which could lead to the person taking on too many tasks. Potentially it put an unsustainable burden on one individual.

*“What happens if our rehab coordinator breaks her leg and is off sick for three months? We would be really vulnerable because we don’t have anyone else who could take over (…)”* Interview 7.

Several participants described the amount of work tasks as undefined and that the function was unlimited but the available time to fulfil the role was not. The managers were aware of these organizational challenges and emphasized their own responsibility to limit the workload and decrease the vulnerability when developing the formulation of the rehabilitation coordinators task and how much time the coordinator can use for it.

*“…to make it work*,* so she won’t fall apart (…) before it was all spread out and everyone pulled her based on both of her professions*,* and that can be quite devastating to feel you can’t keep up.”* Interview 2.

### Change takes time

First-line managers expressed that they were in middle of a process of change. This was in general experienced as joyful and exiting, even if the process took time. The rehabilitation coordinator is a relatively new function in health care, where guidelines are somewhat sparse, and longer experiences are lacking. The informants did not experience clear and manifest guidelines from the health care organization, instead rather vague suggestions about how the function could be arranged. The managers requested such guidelines but had reconciled with constructing the new function together with the rehabilitation coordinator him- or herself. It was emphasized that the assignment was developed in a dialogue, based on frequent meetings. Even though guiding was requested, the first-line managers appreciated the possibilities to influence how the assignment was settled at the health care unit. They preferred the flexibility and the autonomy at the unit, over being controlled in detail.

Time was considered as a lacking resource: both for planning but also to be allocated for the assignment. To settle a new function in health care requires time and the assignment itself must be allocated sufficient time. The intention was to settle a sustainable foundation for the function that also included prevention of sick leave. This was hard to achieve and often it ended up in emergency needs. In general, the function grew over time and the different work tasks seemed to increase all the time. A concept for success was to schedule time for communication between physicians and rehabilitation coordinators. This had improved the internal collaboration.

*“Make sure they get time allocated for it. To be able to work with it and be able to immerse (…) so that one does not have to start all over again on square one every time. So*,* it is important that you set aside time for it and that you formalize the time around it. So that you won’t get an extra workload on top of your regular role*,* instead you need to set aside time for this specific task.”* Interview 2.

The managers engagement and presence were important when forming the assignment, but it was also made clear that the rehabilitation coordinator was a very independent person who took their own initiative. It would have been beneficial to have been supported with guidance about how much time that was needed for rehabilitation coordination in relation to the number of patients. Since this was not available, the managers used frequent evaluations together with the rehabilitation coordinator. This provided information around the need to increase or decrease the time allocated for the assignment.

*“So me and her has brought us into this development together and wobbled our way forward before we found a model we believe in”* Interview 8.

### Need for strengthen status and legitimacy

The rehabilitation coordinator is a role and not a professional title, and the managers actively worked to strengthen the role’s position at the workplace. The specialized study program was described as an opportunity to increase the status and legitimacy of the rehabilitation coordinator. The informants compared rehabilitation coordinators with or without the education and saw that the one-year study program made it possible for the rehabilitation coordinator to perform the most qualified work tasks, not only being an administrative resource that supported with booking appointments.

*“I think my most important task is to support the rehabilitation coordinator in the work she is going to do and stand behind it. To give legitimacy to do her job. (…) to then let her inform about her role*,* give her time out of our meetings*,* that’s the way for me to show her importance. “*Interview 8.

*If you just have the formal skills where you have gained so much*,* you will find and you will see a need*,* which you sometimes may not have known existed.”* Interview 9.

All managers talked about the benefits of having a well-educated rehabilitation coordinator but that it was difficult to reward those benefits adequately in relation to their financial responsibilities. It was good that the health care region supported all health care units for this education economically, but this was actually not a reason for allowing the rehabilitation coordinator to participate in the education. Not being able to compensate for the extended responsibility was experienced as unsatisfying.

Legitimacy also increased when the rehabilitation coordinator had received an expanded network and became more of an expert in the field. This in turn contributed to the whole workplace in a positive way. Education could also serve as an incentive for the rehabilitation coordinator him/herself. It could contribute to better self-esteem and being proud of the assignment. Several informants expressed the opinion that the rehabilitation coordinator should be a specific profession, rather than an assignment and a full-time education should be required.

*“She is much more driven*,* that she has received her diploma and even if you don’t get any identification*,* you still get a little recognized and that pride also makes her much clearer (…) There is another security behind her since she attended the training”* Interview 5.

The importance of communicating the assignment and function in health care, was emphasized. Other professionals at the health care unit were not always sufficiently informed about what services the rehabilitation coordinator could offer. To provide a safe RTW process it was important to have on-going information about the assignment and how the sparse time for the assignment could be used efficiently. Other professionals needed to be aware of this and how much time that was allocated for the assignment. It was also important to make sure that the rehabilitation coordinators workload was reasonable.

## Discussion

In this interview study the themes illustrate that the managers in general had positive experiences of the function rehabilitation coordinator, but also acknowledged challenges to settle the assignment and economize with sparse resources. Augustsson et al. (2020) showed in a recent study, that managers in general had a positive perception of care managers, which also is a rather new function in health care but have a slightly different assignment. They managers prioritized implementation of care management, but they also acknowledge barriers such as lack of time and high work demands [28]. The managers in the present study described their experiences in a similar way.

The rehabilitation coordinator was experienced as a *saviour* who facilitated the work for most of the stakeholders involved in the RTW process and the key aspect was coordination. In a Canadian interview study, Reussel & Kosny, (2019) identified several barriers in collaboration between health care and system stakeholders. They considered these barriers as impacting the patients RTW process negatively [29]. Kilgour and colleagues (2015) have previous described inflexible collaborations between stakeholders in workers’ compensation systems [30]. The managers’ perception was that rehabilitation coordinators, through communicating with external collaborators, relieve the pressure on physicians, who experience sick-listing as troublesome [6, 7]. Further that the rehabilitation coordinators facilitated the RTW process and decreased the irritation among staff members, which contributed to a better work environment. The managers also experienced the rehabilitation coordinator as a uniting function that has great potential to develop the collaboration in the team. The roles that embrace team perspectives can build bridges between professions that contribute to create a coherent, individualized, and high-quality care [24] Holmlund and co-workers (2020) showed in their study, that teamwork is a facilitator for coordinating RTW process [31]. In our study the rehabilitation coordinator was described as a saviour, and this can be seen both from the perspective of the health care team as well as the patient. If smooth, safe and time adapted RTW processes can be arranged, this is beneficial for the patient.

The managers in this study described the rehabilitation coordinator as a personalized function. They perceived the rehabilitation coordinator’s personal characteristics and strong interest in patients on sick leave as superior to formal competences and the underlying profession was not experienced as important. This could reflect the managers’ perception of the role as unclear and may have an impact how rehabilitation coordinators were recruited and employed. Since there were lack of guidelines and raw models, there is risk that the role, at least partly, is formed on own experiences and preferences and results in a variety of practice. The personalized function could also impact how the assignment was conducted, which could lead to a risk for role overload. Stevenson & Duxby (2019) found in their study that novelty and ambiguity in a role have shown to be common properties associated with different stressors [32].

When the assignment as rehabilitation coordinator was relying on one dedicated person, it made the organization vulnerable. If the rehabilitation coordinator was absent no one did his/her job. This could render in waiting periods for patients and when contacts were not taken at the right time, an unsafe RTW-process. In combination with being a much asked for resource, this might put even more pressure on the rehabilitation coordinator.

Since the assignment often was on part-time, a concurrent situation in relation to the underlying profession could be present. In a recent study Holmlund et al., (2022) reported ethical issues in the RTW coordination with conflicting professional values between the health profession and the assignment as rehabilitation coordinator [33]. To face diverse role overload situations at the same time can be difficult for an employee to cope with and cause stress [32].

An unclear assignment, relying on one individual person that needs to cope with concurrence between main profession and the assignment as rehabilitation coordinator, emphasise the need for management support. Stevenson & Duxby (2019) highlighted in their study that when a role is ambiguous and there is a lack of guidelines how to priorities, manager should address the risk for role overload especially when there is a risk of duration of overload over time [32]. Manager must therefore regulate the workload, show the rehabilitation coordinator appreciation, and delimit their work for them to achieve work balance. The managers in this study seem to be aware of their responsibility for creating a good and balanced work situation but further developed reflections regarding how to fulfil this are spares. In a previous study Elshout et al. (2013) showed that there is a link between managers’ leader style and the satisfaction among employees [34].

Changes take times, but the managers found it rewarding and inspiring to be a part of such changes, implementing a new function in health care. Lack of guidance was seen as a barrier but also an opportunity to develop the assignment together with the rehabilitation coordinator, based on local conditions. This was appreciated. Sparce resources regarding time was described not only for the rehabilitation coordination assignment but also for the managers. The managers found it important to support the rehabilitation coordinator and give them legitimacy at the workplace. To strengthen the coordinators legitimacy, special education is essential and a factor that make the implementation of the function more successful. The rehabilitation coordinators will be more stimulated and engaged if the managers encourage them to take part in the special training offered by the employers or universities. Holmlund and co-workers (2020) interviewed rehabilitation coordinators using an implementation framework guide and found it important to formalize training and education as well as provide organizational prerequisites [31]. This need of formalization emerged also among the managers in this study revealing that the constant demands of care-production limit the time required by the learning process.

For patients and other professionals, the new function rehabilitation coordinator might not be well known or not known at all. The implementation of a new role takes time and requires changes in routines and duties. The managers expressed the importance of contributing with generous and concrete support to relieve production requirements and thus create space for learning. Earlier research has shown [35] that managers need to be supportive, encourage education and invest time in the implementation process to succeed.

The professional categories in healthcare are considered to be quite immobile and the introduction of a new role requires both structural and relational efforts. This is confirmed by Cameron et al. (2014) who argue that managers need to legitimize new skills contributions into a field dominated by medical knowledge [36]. Folkman et al. (2019) showed a risk that the contributions of occupational therapists being overridden in cross-professional work because they do not have an obvious place in the care structure [24]. Occupational therapy knowledge is in many ways similar to a rehabilitation coordinator’s area of expertise because it is multidisciplinary and puts the patient in a context to gain an understanding of their needs. The manager in this study was keen to highlight that the coordinator’s perspective contributed to improving the range of care. The manager plays an important role in ensuring that knowledge is not lost as a result of rigid, hierarchical structures.

Managers are responsible for their employees but also for the care provided at the health care unite. In the Danish interview study mentioned earlier [16] patients experienced the lack of coordination as not being able to find their way in the health care system. A rehabilitation coordinator is a resource who facilitates the possibility of individualized and coordinated interventions in the sick leave process, including contact with the patient’s workplace and support and adaptions in their work environment. Simply having a coordinator is however not enough to enhance the RTW process; how and in what area the coordination takes place also plays an essential role. Recent studies on RTW coordination present diverging results on sick leave and RTW [17–19, 37]. RTW coordination differs depending on the cultural context and the specific interventions included in the rehabilitation, which can lead to different outcomes. With the right support from managers and enough allocated time, rehabilitation coordinators could make the RTW process efficient and in favour for the patients.

To continue to develop knowledge about the RTW process and coordination will contribute to a more sustainable development of the function of the rehabilitation coordinator. This is necessary because there is a tangible risk that the burden and stress, which earlier fell upon the clinicians or patients, is transferred to the rehabilitation coordinator. Meanwhile important actions from managers in health care could be to establish more teamwork, allocate time to the mission or increase the rehabilitation coordinator`s credibility. Teamwork in combination with coordinating functions has been shown to have a positive effect on both quality of care and the work satisfaction among staff [34, 38]. This study contributes to the knowledge and understanding of the managers’ possibilities and challenges when leading and implementing a new function in the health care.

### Strengths and limitations

To strengthen trustworthiness the report of this study is based on the COREQ checklist [25]. The interviews were conducted based on an interview guide, which was developed and tested in a pilot-interview. The interviews lasted for 50–75 min, which indicates that the topic was thoroughly discussed. The analysis process is carefully described, and all four authors have been involved during the whole process to ensure credibility and confirmability. Quotations from the different interviews were selected to confirm that the results are grounded in the data. Trustworthiness is strengthened, due to the lack of relations with the informants and that two researchers with limited pre-understanding in the topic conducted the interviews. The reflexivity process during the analyses limited the risk of personal bias that could be a threat towards trustworthiness.

The contact with the possible informants were formalized, based on the recommendations from the Ethical Review Authority and no relationships are present. All participants were informed about the study, both in written and orally. A possible weakness of the study is the small sample (*n* = 10), but since we received rich data, we believe that the number of informants is sufficient. Another aspect that needs to be considered is that there might be a selection bias, where possibly managers that were more positive regarding the rehabilitation coordinator, decided to participate. Even considering this, we have no reason to believe that participants in this interview study differ from other first-line managers in health care, but transferability must be judged by the reader. Finally, two of the authors were engaged in the one-year study program and this could have impacted the analysis process. By discussing reflexivity continuously, we wanted to prohibit negative dependability.

## Conclusion

The first line managers in this study regarded the function rehabilitation coordinator to be a valuable resource that has a great potential to increase patient safety, improve the RTW process and lighten the heavy burden of sick leave from physicians. Since the function often was occupied by one dedicated professional, there were also a risk for vulnerability. Mangers wanted to increase the knowledge regarding the function and thereby strengthen the legitimacy in health care, but such changes took time. The result can serve as a guidance for managers in implementation and support of the function rehabilitation coordinator.

## Supplementary Information

Supplementary Material 1.

Supplementary Material 2.

## Data Availability

The data that support the findings cannot be shared openly due to the privacy of the participants but are available from the corresponding author upon reasonable request.

## Abbreviations

COREQ: Consolidated criteria for Reporting Qualitative research
PHC: Primary Health Care
RTW: Return to work
