# Supplementary material for: Rehabilitation coordinator – managers’ experiences of a new function in health care
Source: BMC Health Serv Res. 2024 Nov 9;24:1371. doi: 10.1186/s12913-024-11856-6 (PMC11549758; doi:10.1186/s12913-024-11856-6)
Supplement: Supplementary file 2 — Supplementary Material 2. [file 12913_2024_11856_MOESM2_ESM.pdf]

Interview Guide

The ~~primary~~ health care center

What does the workplace you are responsible for look like (number of employees, number of people listed at the primary care centre, type of area, teamwork, etc.)?

Can you describe how the rehabilitation coordinator works at your unit? (time allocated, work tasks, assignment, collaboration etc)

How do your assignment and role as a manager look like? (Where are you in the overall organization? Management and other professions?)

Do you have a sick leave policy at the primary care center?

The rehabilitation coordinator function

How do you perceive the rehabilitation coordination function?

What are your experiences of the rehabilitation coordinator function?

What do you know about the special training that the rehabilitation coordinator has undergone?

What was your reason to assign the rehabilitation coordinator to attend this training?

How did you organize the work with sick leave issues before you had a rehabilitation coordinator?

How is it organized today?

What does the rehabilitation coordinator's mission look like today? How is it designed?

How do you evaluate the rehabilitation coordinator’s assignment?

Leadership in relation to the rehabilitation coordinators assignment

How do you view your role as a manager in sick leave issues?

How do you act as a manager of the rehabilitation coordinator? (Support, protect, delimit, new assignment?)

In what way did you support the implementation of the rehabilitation coordinators assignment after the training?

How do you think other employees at the workplace view the function of the rehabilitation coordinator?

How important do you perceive your role as a manager to be in relation to the work of the rehabilitation coordinator and the sick leave process?

What are your expectations and/or concerns for the future when thinking about the function of rehabilitation coordination and sick leave matters in general?
